# Evidence for cerebello-thalamo-cortical hyperconnectivity as a heritable trait for schizophrenia

**DOI:** 10.1038/s41398-019-0531-5

**Published:** 2019-08-20

**Authors:** Hengyi Cao, Martin Ingvar, Christina M. Hultman, Tyrone Cannon

**Affiliations:** 1 Department of Psychology, Yale University, New Haven, CT USA; 2 Department of Clinical Neuroscience, Karolinska Institutet, Stockholm, Sweden; 3 Department of Medical Epidemiology and Biostatistics, Karolinska Institutet, Stockholm, Sweden; 4 Department of Psychiatry, Yale University, New Haven, CT USA

**Keywords:** Schizophrenia, Clinical genetics

## Abstract

Our recent study has demonstrated that increased connectivity in the cerebello-thalamo-cortical (CTC) circuitry is a state-independent neural trait that can potentially predict the onset of psychosis. One possible cause of such “trait” abnormality would be genetic predisposition. Here, we tested this hypothesis using multi-paradigm functional magnetic resonance imaging (fMRI) data from two independent twin cohorts. In a sample of 85 monozygotic (MZ) and 52 dizygotic (DZ) healthy twin pairs acquired from the Human Connectome Project, we showed that the connectivity pattern of the identified CTC circuitry was more similar in the MZ twins (*r* = 0.54) compared with that in the DZ twins (*r* = 0.22). The structural equation modeling analysis revealed a heritability estimate of 0.52 for the CTC connectivity, suggesting a moderately strong genetic effect. Moreover, using an independent schizophrenia cotwin sample (10 discordant MZ cotwins, 30 discordant DZ cotwins, and 32 control cotwins), we observed a significant linear relationship between genetic distance to schizophrenia and the connectivity strength in the CTC circuitry (i.e., schizophrenia MZ cotwins > schizophrenia DZ cotwins > control twins, *P* = 0.045). The present data provide converging evidence that increased connectivity in the CTC circuitry is likely to be a heritable trait that is associated with the genetic risk of schizophrenia.

## Introduction

Psychotic disorders such as schizophrenia possess a strong genetic basis. Empirical data from research on twins and relatives have revealed an estimated heritability of ~80% and a 10-fold increase of risk in first-degree relatives of patients^1,2^, making the study of genetic mechanisms critically important to understand such complex disorders. At the neural level, the genetic risk of schizophrenia embodies the dysregulation of behavior-specific neural circuits, which in turn increases the risk of developing corresponding behavioral defects^3^. Such neurobiological changes (so-called “intermediate phenotypes”^4^ or “endophenotypes”^5^) have offered a valuable avenue to understand the pathogenesis of psychosis and to probe for effective prediction and intervention strategies.

Our recent study has demonstrated that increased connectivity in the cerebello-thalamo-cortical (CTC) circuitry is a state-independent “trait” alteration that may potentially predict the onset of psychosis and distinguish the patients from the healthy population^6^. Such alteration can be reliably detected across multiple functional magnetic resonance imaging (fMRI) paradigms in individuals both at clinical high risk (CHR) and with chronic illness, suggesting a “trait” brain functional abnormality that is robustly present across the entire course of psychotic disorders. These findings highly suggest that increased connectivity in the CTC circuitry may be caused by genetic predisposition to psychosis.

In this study, we tested this hypothesis using two independent twin data sets with multi-paradigm fMRI data. The first data set was part of the Human Connectome Project (HCP, ref. ^7^), including a total of 85 monozygotic (MZ) healthy twin pairs and 52 dizygotic (DZ) healthy twin pairs. Using this sample, we examined the heritability of the connectivity of the CTC circuitry using structural equation modeling (SEM). The second data set was drawn from the Swedish Schizophrenia Twin Study (STAR, ref. ^8^), which included 40 schizophrenia discordant cotwins (10 MZ, 30 DZ) and 32 healthy control twins. We specifically tested the relationship between genetic distance to schizophrenia and the CTC connectivity in this sample. We expected that the CTC connectivity is heritable in healthy twin pairs and is associated with degree of genetic risk of schizophrenia in schizophrenia cotwins (schizophrenia MZ cotwins > schizophrenia DZ cotwins > control twins).

## Methods

### Subjects

Two independent twin data sets were used in this study: the HCP twin sample^7^ and the STAR sample^8^. The HCP data included a total of 137 healthy twin pairs that have completed all eight fMRI paradigms used in the project (resting state, gambling, emotional processing, motor, working memory, relational processing, language processing, and social cognition) and have genotyping data available. Among these twin pairs, 85 were MZ (age 29.32 ± 3.39 years, 70 males) and 52 were DZ (age 28.50 ± 3.50 years, 52 males). The STAR sample comprised of 40 schizophrenia discordant cotwins (10 MZ, age 43.79 ± 11.94 years, 7 males; 30 DZ, age 51.11 ± 10.07 years, 16 males) and 32 healthy control twins (46.37 ± 6.92 years, 14 males), each assessed with a battery of two fMRI paradigms (working memory, emotional processing). The zygosity in both data sets was determined by genotyping data from subjects’ blood samples. The STAR participants were recruited through the Swedish Twin Registry and were evaluated by psychiatrists using the Structured Clinical Interview for DSM-IV^9^. The exclusion criteria for control twins included prior history of neurological or psychiatric disorders, head injury, substance dependence in the last 6 months, mental retardation, and inability to read or comprehend Swedish. All schizophrenia discordant cotwins were free of any psychotic disorders according to the DSM-IV criteria. The participants provided written informed consents and the research protocol was approved by the Regional Ethical Review Board in Stockholm. For sample details, see Tables 1 and 2.

**Table 1:** Demographic data for the HCP sample

	Healthy MZ (*N* = 170, or 85 pairs)	Healthy DZ (*N* = 104, or 52 pairs)	*P* value
Age (year)	29.32 ± 3.39	28.50 ± 3.50	0.06
Sex (M/F)	70/100	52/52	0.15
Head motion (FD, mm)	0.17 ± 0.05	0.16 ± 0.05	0.10

**Table 2:** Demographic and clinical data for the STAR sample

	Schizophrenia MZ cotwins (*N* = 10)	Schizophrenia DZ cotwins (*N* = 30)	Control twins (*N* = 32)	*P* value
Age (year)	43.79 ± 11.94	51.11 ± 10.07	46.37 ± 6.92	0.04
Sex (M/F)	7/3	16/14	14/18	0.34
Head motion (FD, mm)	0.13 ± 0.06	0.13 ± 0.07	0.10 ± 0.05	0.06
DSM Diagnosis	4 major depressive disorder, 1 acute stress disorder, 2 healthy, 3 unavailable	6 major depressive disorder, 2 drug or alcohol dependence, 11 healthy, 11 unavailable	–	–

Note that all schizophrenia cotwins are free of any psychotic disorders

### Imaging paradigms

A total of eight fMRI paradigms were employed in the HCP project. For details on these paradigms see previously published work^10,11^. Notably, the resting-state paradigm was scanned with four sessions spanned across two consecutive days, and the other paradigms were scanned with two sessions on a single day. To balance the amount of data across paradigms and to avoid overrepresentation of resting state in the following cross-paradigm computations, we only used the resting-state data from the two sessions acquired on the first day. The STAR data set used two fMRI paradigms that have been described previously^12,13^. In brief, the spatial working memory task used an event-related design where subjects were asked to encode the location of three target circles and to indicate whether the ensuing probe circle appeared at the same location as one of the target circles. During the block-designed emotional face processing task, subjects were shown a series of emotional faces and geometric shapes and were instructed to match either the target faces or the target shapes.

### Data acquisition

The HCP data were collected from a customized 3T Siemens Connectome scanner equipped with a 32-channel head coil. BOLD images were acquired using a multiband GRE-EPI sequence with the following parameters: TR = 720 ms, TE = 33.1 ms, FA = 52°, FOV = 208 × 180 mm^2^, 2 mm slice thickness, 72 slices, multiband factor = 8. Of note, unlike the commonly applied phase-encoding directions along the “anterior-posterior” axis, the HCP fMRI data were acquired using left-right and right-left phase-encoding directions to accelerate scan time and to minimize image distortion^11^. Each paradigm was scanned with two sessions, with each direction per session. In addition, T1-weighted and T2-weighted images were acquired using 3D-MPRAGE and 3D-T2-SPACE sequences with 0.7 mm voxel size and 224 × 224 mm^2^ FOV. The T1 images used a TR of 2400 ms and TE of 2.14 ms, and the T2 images used a TR of 3200 ms and TE of 565 ms.

The STAR data were acquired from a 1.5 T GE scanner equipped with a standard 8-channel head coil. GRE-EPI sequence was used for functional imaging: TR = 2500 ms, TE = 40 ms, FA = 90°, 3.5 mm slice thickness, voxel size 3.44 × 3.44 × 4.5 mm^3^. T2-weighted images were scanned using a spin-echo sequence with 4000 ms TR, 82 ms TE, and 4 mm slice thickness.

### Data preprocessing

The HCP data were downloaded after the completion of imaging preprocessing (https://db.humanconnectome.org). The preprocessing procedure followed the standard HCP preprocessing pipeline including five major steps: PreFreeSurfer, FreeSurfer, PostFreeSurfer, fMRIVolume, and fMRISurface^14^. In brief, images were corrected for gradient nonlinearity induced distortion, head motion, and phase-encoding related distortion, registered to individuals’ T1-weighted images, and normalized to the MNI space. The STAR data were preprocessed using the standard pipeline implemented in the Statistical Parametric Mapping software (SPM12, https://www.fil.ion.ucl.ac.uk/spm/software/spm12/), including slice timing, head motion realignment, functional-structural image coregistration, MNI space normalization, and spatial smoothing. All preprocessed images were further scrutinized for head motion using the measure of frame-wise displacement (FD^15^) to ensure data quality. All subjects in both data sets had a mean FD < 0.5 mm.

### Cross-paradigm connectivity and extraction of CTC network

**Fig. 1:**
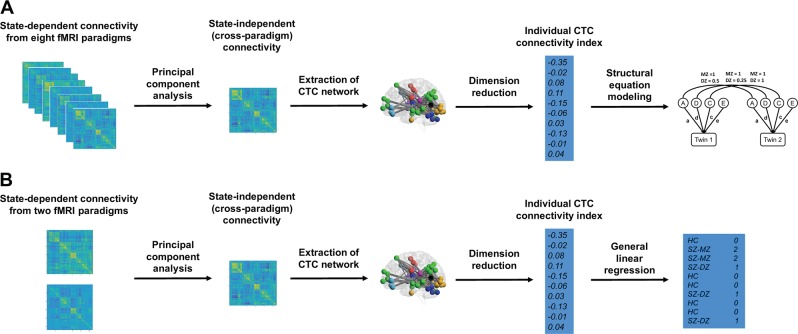
Data processing pipelines for two data sets (**a**: HCP sample; **b**: STAR sample)

The overall processing pipeline of this study is presented in Fig. 1. The cross-paradigm connectivity (CPC) computation and extraction of CTC network followed our prior work^6^. Specifically, the mean time series for each of the 270 nodes in the expanded Power brain atlas^6,13,16^ were extracted from the preprocessed images. The extracted time series were further corrected for task-related coactivations (for task data), white matter and cerebrospinal fluid signals, the 24 head motion parameters (i.e., the six rigid-body parameters generated from the realignment step, their first derivatives, and the squares of these 12 parameters^17,18^), and the FD. The corrected time series were then temporally filtered (task data: 0.008 Hz high pass, rest data: 0.008–0.1 Hz band pass) to account for scanner and physiological noise. Subsequently, a 270 × 270 pairwise connectivity matrix was generated using Pearson correlation for each subject during each paradigm and scan session.

Following prior suggestions^11^, we averaged the derived connectivity matrices across the two sessions for each paradigm to boost signals, generating eight paradigm-specific connectivity matrices for each subject in the HCP data and two paradigm-specific connectivity matrices for each subject in the STAR data. The matrices for each subject were vectorized, concatenated across paradigms, and then decomposed into a set of principal components (PCs) using singular value decomposition (SVD). The first PCs generated from the analysis represented the shared CPC and were thus extracted. From these CPC matrices we further extracted the identified CTC network based on our prior work^6^.

### Heritability estimation of CTC connectivity

The HCP data were employed to quantify heritability of the CTC connectivity. We first calculated the similarities of the MZ and DZ twin pairs using intra-class correlations. A larger correlation coefficient between MZ pairs compared with that between DZ pairs indicates a potential genetic effect. Since the MZ correlation was larger than twice of the DZ correlation in our sample (see Results section), we estimated an ADE model using structural equation modeling (SEM). This is due to the fact that dominant genetic effects (D) would decrease the DZ correlation relative to the MZ correlation^19,20^. Specifically, using the SEM toolbox OpenMx implemented in R (https://openmx.ssri.psu.edu/), we decomposed the total variance of phenotype into additive genetic effects (A), dominant genetic effects (D), and unique environmental effects (E). In general, the MZ twins share 100% of additive and dominant genetic effects, while the DZ twins share 50% and 25% of additive and dominant genetic effects, respectively^19,20^. Based on this information, we estimated the squares of each path coefficients (*a*^2^*, d*^2^*, e*^2^) that reflect the proportion of phenotypic variation attributable to the A, D, E effects, respectively. This was done by iteratively maximizing the log-likelihood between observed and predicted covariance matrices. The goodness-of-fit of the estimated model was quantified by −2 times the log-likelihood (−2LL, ref. ^19^) as well as the Akaike Information Criterion (AIC, ref. ^21^), and was compared to the saturated model in which all covariances were treated as free parameters using Chi-squared test. A non-significant *P* value ( > 0.05) indicates a good model fit. Moreover, the full ADE model was further compared with nested AE, DE, and E models, and the most parsimonious model was determined by the one with the smallest −2LL and AIC values. Heritability (*h*^*2*^) was subsequently quantified as the proportion of phenotypic variance attributable to genetic effects in the most parsimonious model.

### Association of CTC connectivity with schizophrenia genetic risk

We examined the association between CTC connectivity and genetic risk of schizophrenia in the STAR data. Specifically, a linear regression model was employed where CTC connectivity was included as dependent variable and genetic proximity to schizophrenia (i.e., schizophrenia MZ cotwins > schizophrenia DZ cotwins > control twins), age, sex, and FD were included as regressors. A significant regression coefficient for genetic proximity indicates that CTC connectivity is associated with schizophrenia genetic risk. Since the size of STAR sample was relatively small, we further included all twins in the HCP sample as controls in the regression model. To control for discrepancy between the two data sets, cohort was included as an additional regressor in the model for the combined sample.

## Results

### Heritability of CTC connectivity

**Fig. 2:**
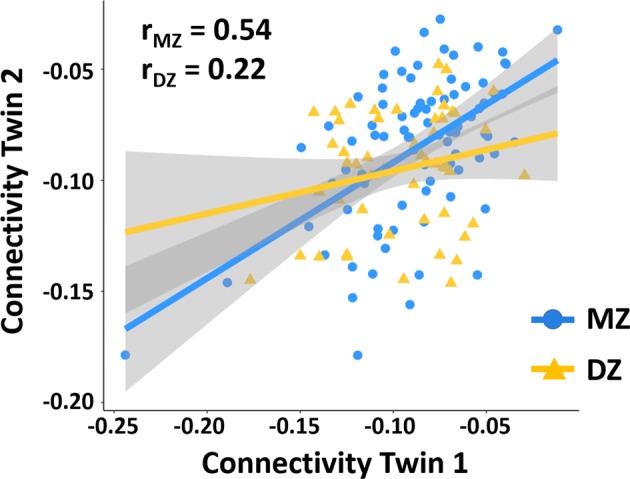
Intra-class correlations of CTC connectivity among the MZ and DZ twins in the HCP data. The correlation coefficient was much higher for the MZ twins compared to that for the DZ twins, suggesting a potential genetic effect

There were no significant differences in the mean and variance of CTC connectivity between MZ and DZ twins in the HCP sample (*P* = 0.12 and 0.46, respectively). The intra-class correlation coefficients for CTC connectivity among MZ and DZ twin pairs in the HCP sample were 0.54 and 0.22, respectively, suggesting a genetic effect (Fig. 2). Since the correlation between the MZ twins was larger than twice of that between the DZ twins, we evaluated an ADE model using SEM. The results revealed a heritability of 0.52 (95% CI: 0.36–0.64). Specifically, 52% of the variance in CTC connectivity was attributable to additive genetic effects (*a*^2^ = 0.52), and 48% was attributable to unique environmental effects (*e*^2^ = 0.48). In contrast, dominant genetic effects were estimated to be negligible (*d*^2^ < 0.001). There was no significant difference in comparison between the ADE model and the saturated model (*−2LL*_*ADE*_ = 1388.18, *AIC*_*ADE*_ = 848.18, *−2LL*_Saturated_ = 1379.74, *AIC*_Saturated_ = 851.74, *P* = 0.2), indicating a good model fit for the present data. Moreover, the further comparison of the full ADE model with the nested models showed the smallest −2LL and AIC for the AE model (*−2LL*_*AE*_ = 1388.18, *AIC*_*AE*_ = 846.18, *P* = 1), suggesting that the AE model is the most parsimonious and should be accepted as the model that best fits the data.

### Association of CTC connectivity with schizophrenia genetic risk

**Fig. 3:**
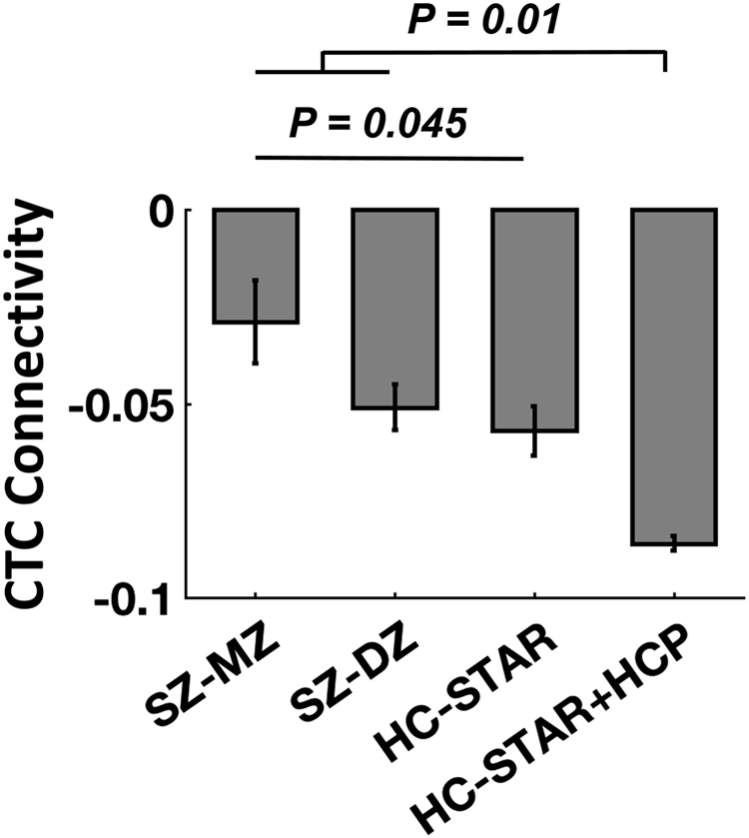
Linear regression model showed significant linear relationship between CTC connectivity and genetic proximity to schizophrenia (i.e., schizophrenia MZ cotwins > schizophrenia DZ cotwins > healthy controls), suggesting that CTC hyperconnectivity is associated with genetic risk of schizophrenia. Error bars stand for standard errors

The Levene test showed an equal variance of CTC connectivity between the three examined groups (*P* = 0.84). The regression analysis revealed a significant linear relationship between genetic proximity to schizophrenia and CTC connectivity in the STAR sample (*P* = 0.045, Fig. 3), suggesting that CTC connectivity is related to schizophrenia genetic risk. In particular, schizophrenia MZ cotwins showed the highest connectivity, followed by schizophrenia DZ cotwins, while control twins had the lowest connectivity, which is consistent with our prior findings that patients with schizophrenia are associated with higher connectivity in the CTC circuitry^6^. Moreover, the inclusion of HCP data as control subjects demonstrated even stronger effect (*P* = 0.01), which further supports the idea that CTC hyperconnectivity is likely to be a heritable trait that is associated with genetic predisposition to schizophrenia.

### Supplementary analysis on head motion

Previous study has reported that head motion is moderately heritable during resting-state fMRI scans^22^. As a result, it is possible that our results may be influenced by this confounding factor. To test this hypothesis, we further examined the intra-class correlation coefficients for head motion FD measures across the MZ and DZ twins, respectively, in the HCP sample. We found that the correlation coefficients for MZ and DZ twins in the current sample were very close (*r*_MZ_ = 0.38, *r*_DZ_ = 0.34), suggesting a very trivial genetic effect. This supplementary analysis indicates that the heritability in CTC connectivity is unlikely to be driven by the heritability in head motion.

### Supplementary analysis on ACE model

In this study, an ADE model was chosen to estimate heritability since the correlation of CTC connectivity between the MZ twins was larger than twice of that between the DZ twins. In a supplementary analysis we also estimated heritability using an ACE model. The ACE model showed a heritability of 0.38 (95% CI: 0.17–0.56). In particular, similar to the results from an ADE model, 38% of total variance was attributable to additive genetic effects (*a*^2^ = 0.38), 62% was attributable to unique environmental effects (*e*^2^ = 0.62), and common environmental effects were negligible (*c*^2^ < 0.001). This supplementary analysis again suggests that the variations in CTC connectivity are mainly caused by additive genetic effects and unique environmental effects.

### Supplementary analysis on statistical power

Because the STAR sample in this study was relatively small, we performed a power analysis to determine whether the study was adequately powered to detect the observed effect sizes. The power to reject the null hypothesis of no group difference was 0.7 for the comparison between SZ-MZ cotwins and SZ-DZ cotwins (*α* = 0.05, one-tailed test). The power increased to 0.9 when comparing SZ-MZ cotwins and HC twins (*α* = 0.05, one-tailed test), suggesting that the present sample, although small, is likely to have been adequately powered.

## Discussion

This study tested the hypothesis that the CTC hyperconnectivity is related to genetic predisposition to schizophrenia using two independent twin data sets. Overall, we observed a relatively high heritability of CTC connectivity in the HCP twin sample and a significant association between CTC hyperconnectivity and genetic proximity to schizophrenia. These findings provide converging evidence that CTC hyperconnectivity is likely to be a heritable trait that is related to the genetic risk of schizophrenia.

Given that increased connectivity in the CTC circuitry is a “trait” abnormality that can be reliably detected across different behavioral domains in psychosis, the strong relationship between this change and genetic risk of schizophrenia is within expectation. Notably, both the structure and the function of two key brain regions in this circuitry, the thalamus and cerebellum, have previously been found to be heritable^23–29^ and potentially related to schizophrenia genetic risk^30,31^. Several cortical functional systems involved in this circuitry, including the default-mode network, fronto-parietal network, and attentional network, were also found to be heritable in the healthy population^32–34^. Here, our findings parallel with these previous findings and further demonstrate that a widely distributed network covering cerebellum, thalamus and cerebral cortex is heritable and regulated by genetic risk of schizophrenia.

Since CTC hyperconnectivity may reflect a downstream phenomenon of N-methyl-D-aspartate receptor (NMDAR) deficits^35–37^, this alteration is presumably related to genes that are associated with glutamate and/or gamma-Aminobutyric acid (GABA) signaling. Large-scale genome-wide association studies (GWAS) have identified a set of common genetic variants in glutamatergic genes in patients with schizophrenia, including, for instance, *GRM3, GRIN2A, GRIA1, SRR, CLCN3*, among others^38,39^. Gene expression and brain imaging analyses suggested a converging effect of these genes on brain structure and function, especially in the prefrontal cortex^40–43^, hippocampus^43^, and thalamus^43^. Moreover, pathway analysis also revealed a significant association of genetic variants in NMDAR complex and glutamate metabolism pathways with the pathogenesis of schizophrenia^44,45^. In addition to common variants, rare genetic mutations such as copy number variation have also been found to be highly enriched for genes involved in the glutamatergic and GABAergic neurotransmission^46–48^. These lines of evidence suggest that neurotransmission-linked schizophrenia genetic factors may affect excitation–inhibition balance in the human brain that further sets an over-excitatory tone for the overall brain connectivity. Such altered brain connectivity, especially in a CTC circuitry, is therefore presented as a “trait” abnormality that can be reliably detected across various states and phases of schizophrenia.

Other genes that may relate to the connectivity in the CTC circuitry are those associated with dopamine signaling, given the essential role of this circuitry in error processing^49,50^. Notably, dopamine system plays a key role in error processing in the brain. It is assumed that the prediction errors, which reflect the mismatch between the priors and outcomes, are encoded by the phasic dopamine neuron firing in the ventral-tegmental area (VTA) of the midbrain^51,52^. The activity of the VTA dopamine neurons alters the dopamine input to the mesolimbic and mesocortical pathways, which in turn, modulates a variety of human behaviors such as cognition, reward processing, reinforcement learning, and social activity^53–55^. Previous studies have shown that dopamine-related genes such as *DRD2, DRD4, COMT*, and *DAT* influence error-related negativity^56–58^ and increase the risk for schizophrenia^38,59^. As a result, genetic variants involved in dopamine signaling may regulate error processing associated behaviors via the regulation of the CTC circuitry.

One major limitation of our study is the relatively small sample size, especially for schizophrenia cotwins. Although we showed that this small sample is still likely to have reasonable statistical power, further fMRI studies with larger twin samples are encouraged to replicate these findings. Second, different fMRI paradigms were used in two studied samples. While including different paradigms would strengthen the argument that CTC hyperconnectivity is a heritable “trait” rather than a “state-dependent” change, such differences may to certain degree influence the outcome individual “trait” networks between samples. Third, while we only included schizophrenia discordant cotwins without psychotic disorders in this study, some of the cotwins were diagnosed with other types of mental disorders (in particular major depressive disorder, see Table 2). As a result, our findings may to certain degree be affected by this confounding factor. Last but not least, while we provided direct evidence that CTC hyperconnectivity is related to the genetic risk of schizophrenia and discussed potential associations between such phenotype and neurotransmitter signaling, how common variants in glutamate and dopamine-related genes would affect the connectivity in the CTC circuitry is still an open question. Future imaging genetic studies are encouraged to investigate such relationships.

In sum, using multi-paradigm fMRI data from two independent twin cohorts, our study shows the first evidence that increased connectivity in the CTC circuitry is likely to be a heritable trait related to the genetic risk of schizophrenia. These findings echo our previous work on CTC hyperconnectivity as a biological trait for psychosis prediction and overall highlight the critical role of CTC circuitry in the pathogenesis of schizophrenia.
